# Fully controllable multichannel waveguides induced by counterpropagating Bessel beams

**DOI:** 10.1038/s41598-022-22384-w

**Published:** 2022-10-20

**Authors:** Yue Chai, Nicolas Marsal, Delphine Wolfersberger

**Affiliations:** 1 Université de Lorraine, CentraleSupélec, LMOPS, F-57070 Metz, France; 2 Chair in Photonics, CentraleSupélec, LMOPS, F-57070 Metz, France

**Keywords:** Nonlinear optics, Solitons

## Abstract

We theoretically analyze the waveguiding structures photo-induced by two incoherent counter-propagating Bessel beams (BBs) in a biased photorefractive crystal. We demonstrate that the cross-coupling of two BBs enables adressable channels and tunability of the forming guiding structures. The truncation parameter of the BBs, their Bessel orders and the misalignment between the two beams are all key parameters for tailoring the characteristics of the photo-induced waveguides such as the number of outputs, the output intensity levels and the distance between each output channel. Accordingly, we optimized the different parameters for designing not only a fully tunable Y-coupler but also optical splitters with up to five outputs and even more complex star couplers for all-optical interconnect applications. Finally, we report on the stability behavior of the photo-induced platform. The stability threshold depends on the nonlinearity parameter beyond which the beams display time-periodic, quasi-periodic and turbulent dynamics where spatially localized instabilities can be observed. All these results suggest more opportunities for fully controllable complex waveguiding structures and new all-optical solutions for active components in optical telecommunication and innovative ways of performing optical computing based on spatiotemporal chaos.

## Introduction

Optical interconnection technologies are expected to replace electronic systems for improving information processing performance due to their broad bandwidth, high speed, long-distance data transmission and potential reconfigurability^1,2^. Current optical devices, such as optical fibers, silicon waveguides, and photonic integrated circuits, are limited because those classical optical systems necessarily work with high peak power and are usually bulky, passive, and not reconfigurable^3^. Another widespread approach is based on scalable and reconfigurable optical techniques using Kerr or photorefractive (PR) medium. The latter is more appealing because of its low power requirements^4^ and its waveguide stability in 2D^5^. In a PR crystal, an incident light beam may photo-excite free charge carriers, which are then diffused and redistributed to finally create a space-charge field. This internal field modifies the refractive index distribution through the Pockels effect in the PR medium^6^. The light can therefore be confined and propagates inside the photo-induced refractive index structures, giving rise to all-optical waveguiding channels and switching devices^7^. In addition, thanks to the unique characteristic of PR crystals, it is easy to erase the photo-induced waveguides by launching an incoherent white light and address new configurations. Gaussian beams were the first and the most commonly used optical beams for photo inscriptions^8–10^. In recent years, unconventional beams, such as Airy beams, were also studied for photo-inducing complex waveguiding structures in all-optical routing applications due to their multi-lobes profiles and self-accelerating characteristic^11–15^.

Similarly, Bessel beams (BBs), another unconventional beam proposed by Durnin in 1987, have also been extensively studied for their non-diffracting and self-healing properties^16^. Due to its mathematical definition, a BB possesses an infinite energy. Thanks to a truncation parameter (Gaussian term), BB can therefore be experimentally generated for example by using axicon or Spatial Light Modulators (SLM)^17^. Depending on its order, the BB intensity profile shows a central bright or dark spot and several surrounding rings^18^. Such symmetrical multi-lobes profiles and its non diffractive property make BB interesting for a variety of applications: free-space optical interconnects^19^, switches in the turbulent atmosphere^20^, light localization by inducing Bessel photonic lattices^21^.

BBs may also present self-trapping and breather-forming behavior when propagating in nonlinear media^22^. Due to focusing phenomena, several integrated optical components, such as reconfigurable soliton networks^23^, multiport splitters^24^, and coupling strength tunable optical couplers^25^, have been created by multiplexing co-propagating diffraction-free BB in Kerr or PR crystal. Recently, we demonstrated that a single diffracting BB can also induce various waveguiding structures with multiple channels in a biased PR crystal, which can be tailored by different parameters such as truncation, nonlinearity, beam order, and beam size^26^. Despite this reconfigurability, the guiding properties of these photo-induced configurations are still limited by the chosen profile (order) of the BB. By contrast, the counter-propagating (CP) collision of two mutually incoherent beams gives rise to the cross-coupling of the beams’ lobes via the combination of the mixing refractive index structures generated inside the propagating medium. The cross-coupling enables more tunable parameters and more complex waveguiding configurations. In addition, the CP scheme also supports a dynamical instability mediated by intrinsic feedback^13,27^, which motivates the active optical components for all-optical routing and switching.

In this paper, we numerically analyze the waveguiding structures photo-induced by two incoherent CP BBs in a biased PR crystal. As already explained, the cross-coupling between two CP beams may generate more guiding channels and addressable outputs. By comparing with previous works based on Gaussian beams^7^, Airy beams^12,14^, and a single BB^26^, we show more complex optical couplers, addressing up to 9 outputs, with larger input-to-output shifts, wider distances between each output, and higher guiding efficiencies. By changing the parameters, such as the truncation of the beam, the transverse shift between the two CP BBs, and the order of each beam, we propose various reconfigurable waveguiding structures. We also demonstrate the flexible control for the number of the outputs, the guiding efficiency of each possible channel, and the distance between each output. In addition, we display and analyze the spatiotemporal dynamics of CP zero-order BBs and CP one-order BBs by varying the nonlinear strength. In both cases, the instability thresholds for the nonlinearity are identified beyond which the interactions lead to time-periodic, quasi-periodic, and turbulent dynamics where spatially localized instabilities can be observed.

## Numerical model

**Figure 1:**
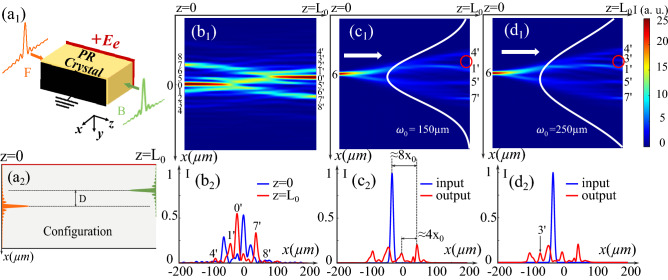
(**a**_**1**_) (**a**_**2**_) Principle scheme for the interaction of two CP BBs in a PR crystal. (**b**_**1**_) Normalized intensity distribution of the CP BBs ($x_0=10$ μm, $\omega _0=150$ μm, $F_0=B_0=\sqrt{25}$) with a transverse distance $D=21$ μm under the nonlinear condition $\Gamma = 3$. (**b**_**2**_) Transverse intensity profiles corresponding to each side of the PR crystal ($z=0,\;z=L_0$). (**c**_**1**_) (**c**_**2**_) Test of the configuration in (**b**_**1**_) with a probe Gaussian beam: (**c**_**1**_) Normalized intensity distribution, (**c**_**2**_) normalized intensity profiles. (**d**_**1**_) (**d**_**2**_) Test of the configuration induced by less truncated CP BBs ($x_0=10$ μm, $\omega _0=250$ μm, $F_0=B_0=\sqrt{25}$) propagating under the same condition ($\Gamma =3$): (**d**_**1**_) Normalized intensity distribution, (**d**_**2**_) Normalized intensity profiles.

The CP interaction scheme of two BBs is illustrated in (Fig. 1a_1_). Both CP BBs propagate along the *z*-axis. The crystallographic *c*-axis of the crystal along which we apply the electric field corresponds to the *x*-axis. As discussed in^28^, the focusing effect in the *c*-axis direction, which is caused by the drift effect in the crystal, is much more intense. Consequently, to achieve waveguiding structures in a bulky 3D volume, the first step is to study the photo-induced configurations in the radial section composed of the c-axis and the direction of propagation (z-axis). For this reason, we restrict the BBs propagation in this plane, and the top view of the crystal in (Fig. 1a_2_) displays our (1+1)D situation. The forward beam *F* and the backward beam *B* are injected respectively to the left ($z=0$) and right ($z=L_0$) sides of the crystal with a transverse distance *D* and propagate in the opposite directions. Assuming that the main lobe of the forward beam is at the transverse position $x=0$, the normalized input profiles of these two 1D BB are mathematically defined by:

1 $$\begin{aligned} F(X,Z=0)= & {} F_0J_{n_1}(X)\exp (-\frac{X^2}{(\omega _0 \cdot k_t)^2}), \end{aligned}$$

2 $$\begin{aligned} B(X,Z=L)= & {} B_0J_{n_2}(X+d)\exp (-\frac{(X+d)^2}{(\omega _0 \cdot k_t)^2}), \end{aligned}$$

where $F_0$ and $B_0$ are the maximum electric field amplitudes of the forward and backward beams. Similar to the numerical model in^26^, *J* is the Bessel function with $n_1$, $n_2$ corresponding to the related orders of the forward and backward beams, $X=k_t x=2x/x_0$ is the normalized transverse coordinate, where $x_0$ is the waist of the main lobe of the zero-order BB, and $k_t$ is the transverse wave number defined by $k_t=2/x_0$. $Z=z/L_d$ and $L=L_0/L_d$ are respectively propagating length and crystal length normalized by $L_d=k x_0^2/2$, which is the Rayleigh length of the separated central lobe of the zero-order BB, $L_0$ is the length of the crystal ($L_0=1$ cm is fixed in the following calculations), and *k* is the wavenumber^29^. $d=D/x_0$ is the dimensionless transverse distance related to *D*, and $\omega _0$ is the waist of the Gaussian truncated term. As we consider two mutually incoherent BB propagating in the opposite directions in the medium, the propagation equations are given by^30^:

3 $$\begin{aligned}&i\partial _Z F +\partial _X ^2 F =\Gamma E_0F, \end{aligned}$$

4 $$\begin{aligned}&-i\partial _Z B +\partial _X ^2 B =\Gamma E_0B, \end{aligned}$$

$\Gamma = \frac{k^2}{k_t^2} n_0^2 r_{eff} E_e$ represents the nonlinearity strength where $r_{eff}$ is the effective component of the electro-optic tensor, $n_0$ is the linear refractive index ($n_0=2.3$ in all following calculations for a SBN crystal), and $E_e$ is the applied external electric field. $E_0$ is the nonlinear response of the crystal defined by $E_0=E_{sc}/E_e$, where $E_{sc}$ is the space charge field formed by photo-excited donors through the drift effect in the PR crystal^6^. The temporal evolution of $E_0$ is given by assuming relaxation-type dynamics^30^:

5 $$\begin{aligned} \tau \partial _t E_0+E_0=-\frac{I}{(1+I)}, \end{aligned}$$

where the relaxation time of the crystal $\tau =\tau _0/(1+I)$ is inversely proportional to the normalized total intensity $I=|F|^2+|B|^2$, and $\tau _0$ is the characteristic response time of the PR crystal.

## Results and discussion

Similar to^12,14,26^, we solve the propagation equations Eqs. (3)–(4) concurrently with the temporal equation Eq. (5) by Fast Fourier Transform Beam Propagation Method (FFTBPM) and numerical Runge-Kutta method RK4. The time loop duration is set to $100 \tau _0$ so that all waveguiding configurations presented before the last paragraph on spatiotemporal dynamics analysis concern the stationary state.

### Control of the output channels number through the beam truncation

Firstly, we consider two diffracting zero-order BB with the same parameters: $x_0=10$ μm, $\omega _0=150$ μm, $F_0=B_0=\sqrt{25}$ misaligned with the transverse distance of $D=21$ μm propagating in the opposite directions under the nonlinear condition ($\Gamma = 3$). Figure 1$\hbox {b}_1$ and $\hbox {b}_2$ respectively show the intensity distribution and the transverse profiles on both sides of the crystal [left side ($z=0$ blue line) and right side ($z=L_0$ red line)]. The numbers (0, 1 to 8) and ($0^{\prime }$, $1^{\prime }$ to $8^{\prime }$) represent the positions of the lobes on each side of the crystal (same for all following waveguiding configurations). As demonstrated in^26^, the side lobes of a single diffracting BB focus and induce two efficient channels forming a Y-coupler during their propagation under the nonlinear condition. The head-on collision of such two incoherent beams results in the cross-coupling of the adjacent branch channels of the Y-couplers photo-induced by the forward and backward beam via the mixing refractive index structure, as shown in (Fig. 1$\hbox {b}_1$). Several configurations depending on *d*, $\omega _0$, $\Gamma$ have been explored. Only the ones presenting the best interest for optical interconnects applications are presented. To test the guiding properties of the different coupling configurations, we fix the photo-induced refractive index distribution and inject a Gaussian probe beam at the position of the forward beam side lobe 6 , which is advantageous for all-optical interconnects. The probe beam propagates linearly along the direction indicated by the white arrow shown in (Fig. 1$\hbox {c}_1$). Same as^26^, we only take into account the outputs of more than $10\%$ of the maximum input probe beam intensity for optical interconnects applications^14^. Figure 1$\hbox {c}_1$ and $\hbox {c}_2$ illustrate the intensity distribution of the test beam and its transverse input (blue line in Fig. 1$\hbox {c}_1$) and output (red line in Fig. 1$\hbox {c}_1$) profiles. Due to the diffraction of the probe beam, it splits firstly into its nearby channel and then into four outputs spatially evenly distributed $(4^{\prime },1^{\prime },5^{\prime },7^{\prime })$. This large number of outputs is favorable for multiplexed all-optical interconnects applications. The distance between two adjacent outputs is nearly $4x_0$, which is large enough to avoid cross-talking and make the outputs well addressable. Besides, we also notice that the photo-induced waveguiding structure achieves a large input-to-output shift $8x_0$ indicated in (Fig. 1$\hbox {c}_2$), which is necessary for the large transverse interconnects distance for all-optical interconnects as discussed in^14^. Unlike self-accelerating beams, waveguiding configurations induced by two straight-propagating beams, such as Gaussian beams, cannot have such a large input-to-output shift because of the spatial limitations. Instead, using such BBs can break through this limit thanks to their diffracting characteristic and multi-lobes profiles.

To further specify how the multi-lobes profiles of BB influence the photo-induced waveguiding structure, we decided to investigate their Gaussian envelope, i.e., the truncation parameter $\omega _0$. We increase it up to $250$ μm for both two incident BB and calculate Eqs. (1)–(5) remaining the other parameters fixed. Then, we inject a probe beam in position 6 (same position as before) of the new photo-induced configuration. Figure 1$\hbox {d}_1$ and $\hbox {d}_2$ depict the intensity distribution and the corresponding input and output profiles. Comparing the white Gaussian envelope in Fig. 1$\hbox {c}_1$ to that shown in (Fig. 1$\hbox {d}_1$), a wider envelope allows the side lobes of BB to carry more energy, i.e., higher intensity. These side lobes can photo-induce higher refractive index modulations which trap more energy. As a result, one more output with $10.7\%$ of the maximum intensity at position $3^{\prime }$ is obtained, as shown in (Fig. 1$\hbox {d}_1$ and $\hbox {d}_2$).

In this section, we have demonstrated that the waveguiding structures induced by two 1D-CP BBs can be used as multichannels splitters with up to five addressable outputs that cannot be achieved by using one single BB. Furthermore, the truncation is an interesting parameter to set the number and the intensity of the outputs.

### Fully controllable “Y-coupler” through the BB transverse shift

**Figure 2:**
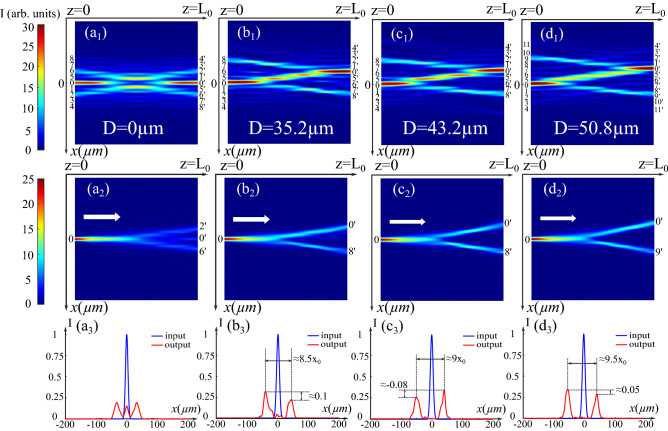
(${\mathbf{a}}_{{\mathbf{1}}}$)–(${\mathbf{d}}_{{\mathbf{1}}}$) Normalized intensity distributions induced by two CP BBs ($x_0=10$ μm, $\omega _0=150$ μm, $F_0=B_0=\sqrt{25}$) with the transverse distance of (${\mathbf{a}}_{{\mathbf{1}}}$) $D=0$ μm (${\mathbf{b}}_{{\mathbf{1}}}$) $D=35.2$ μm (${\mathbf{c}}_{{\mathbf{1}}}$) $D=43.2$ μm (${\mathbf{d}}_{{\mathbf{1}}}$) $D=50.8$ μm under the nonlinear condition ($\Gamma = 3$). (${\mathbf{a}}_{{\mathbf{2}}}$)–(${\mathbf{d}}_{{\mathbf{3}}}$) Test of the waveguiding configurations with the same Gaussian probe beam injected in 0: (${\mathbf{a}}_{{\mathbf{2}}}$)–(${\mathbf{d}}_{{\mathbf{2}}}$) Normalized intensity distributions (${\mathbf{a}}_{{\mathbf{3}}}$)–(${\mathbf{d}}_{{\mathbf{3}}}$) Normalized intensity profiles respectively corresponding to the configuration in (${\mathbf{a}}_{{\mathbf{1}}}$)–(${\mathbf{d}}_{{\mathbf{1}}}$).

According to the previous analysis, in a biased PR crystal, two mutually incoherent CP BBs may attract and overlap providing potential waveguiding structures with more accessible channels. As discussed in the case of Airy beams^15^, the attraction between two beams can be controlled by their misalignment. Thus, to clarify the impact of the attraction of two BBs, we consider the same situation as that described in (Fig. 1$\hbox {b}_1$) and only change the transverse distance *D* between the two CP BBs. Figure 2$\hbox {a}_1$–$\hbox {d}_1$ show their intensity distributions versus the transverse distance *D*. The configuration induced by two aligned BBs in (Fig. 2$\hbox {a}_1$) remains symmetrical along the axis $x=0$. When the probe beam is injected into 0, as depicted on (Fig. 2$\hbox {a}_2$ and $\hbox {a}_3$), it splits into three outputs symmetrical around $x=0$ and located at ($2^{\prime }$, $0^{\prime }$, $6^{\prime }$). Instead, when two CP BBs are misaligned, as shown in (Figs. 2$\hbox {b}_1$–$\hbox {d}_1$), the photo-induced configurations are no longer symmetrical. To analyze their guiding properties, we test all these photo-induced waveguiding structures by injecting the same Gaussian probe beam into position 0 and by propagating them to the (+ *z*)-direction. Figure 2$\hbox {b}_2$–$\hbox {d}_2$ show the normalized intensity distribution in the crystal, and Fig. 2$\hbox {b}_3$–$\hbox {d}_3$ show the corresponding input and output profiles. In all these cases, unlike that in (Fig. 2$\hbox {a}_1$), the incident energy splits into two channels and performs as an asymmetrical Y-coupler. As indicated in (Fig. 2$\hbox {b}_3-\hbox {d}_3$), the output on the side of $x>0$ ($8^{\prime }$ in Fig. 2$\hbox {b}_2-\hbox {c}_2$ and $9^{\prime }$ in Fig. 2$\hbox {d}_2$) remains at the same position, while the other ($0^{\prime }$ in Fig. 2$\hbox {b}_2-\hbox {d}_2$) gradually moves away from it as *D* increases (The distance between two outputs are respectively $8.5x_0$, $9x_0$, $9.5x_0$.). This output ($0^{\prime }$) always follows the main lobe of the backward beam due to the interaction and the photo-induced waveguiding structure created between the two incoherent BBs. Moreover, the output profiles in (Fig. 2$\hbox {b}_2-\hbox {d}_2$) show that the intensity difference between two outputs varies as a function of the transverse distance *D* (respectively 0.1, − 0.08, 0.05). When we vary this parameter, two CP BBs interact differently so that the two channels for the Y-coupler own different ratios of the guiding efficiency depending on their refractive index change distributions.

In summary, as the unique parameter of CP schemes, the transverse distance *D* between two BBs can modulate the outputs number, the distance between the outputs, and the guiding efficiency of the channels. We should emphasize that using two CP BBs allows more flexible control of the guiding properties that is not possible to realize with one BB.

### All-optical routing using multiple inputs

**Figure 3:**
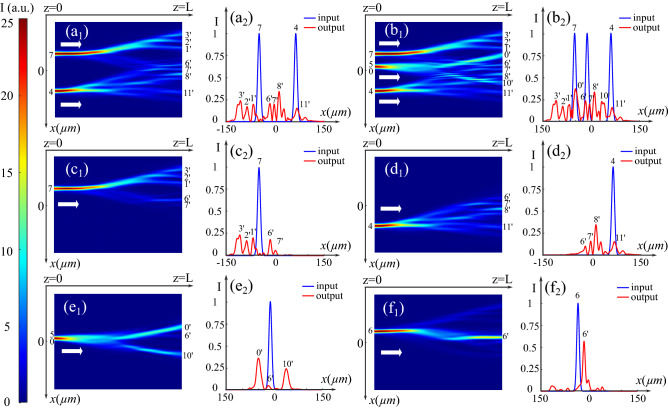
Test of the photo-induced waveguiding structure in (Fig. 2$\hbox {d}_1$) by (${\mathbf{a}}_{{\mathbf{1}}}$) (${\mathbf{a}}_{{\mathbf{2}}}$) two probe Gaussian beams and (${\mathbf{b}}_{{\mathbf{1}}}$) (${\mathbf{b}}_{{\mathbf{2}}}$) three probe Gaussian beams: (${\mathbf{a}}_{{\mathbf{1}}}$) (${\mathbf{b}}_{{\mathbf{1}}}$) Normalized intensity distributions (${\mathbf{a}}_{{\mathbf{2}}}$) (${\mathbf{b}}_{{\mathbf{2}}}$) Corresponding transverse profiles on the left and right side of the crystal. (${\mathbf{c}}_{{\mathbf{1}}}$) (${\mathbf{c}}_{{\mathbf{2}}}$)–(${\mathbf{e}}_{{\mathbf{1}}}$) (${\mathbf{e}}_{{\mathbf{2}}}$) Individual propagation of each probe beam: Intensity distribution and corresponding transverse profiles of the propagation of the probe beam injected in (${\mathbf{c}}_{{\mathbf{1}}}$) (${\mathbf{c}}_{{\mathbf{2}}}$) position 7, (${\mathbf{d}}_{{\mathbf{1}}}$) (${\mathbf{d}}_{{\mathbf{2}}}$) position 4, and (${\mathbf{e}}_{{\mathbf{1}}}$) (${\mathbf{e}}_{{\mathbf{2}}}$) between position 5 and 0. (${\mathbf{f}}_{{\mathbf{1}}}$) (${\mathbf{f}}_{{\mathbf{2}}}$) Intensity distribution and transverse profiles of the propagation of the probe beam injected in position 6.

All photo-induced waveguiding structures discussed above possess multiple potential outputs on each side of the crystal (at least nine), which allow them to meet the requirements for waveguides with multiple inputs-outputs in all-optical interconnects. Therefore, we consider the configuration with the most channels (Fig. 2$\hbox {d}_1$) and test it by propagating simultaneously multiple probe beams. Firstly, as shown in (Fig. 3$\hbox {a}_1$ and $\hbox {a}_2$), we inject two probe beams respectively in (7,4) and obtain seven outputs on the other side of the crystal. To specify the coupling behavior of the two probe beams and analyze the functions of this configuration, we also present the intensity distribution and the corresponding transverse profiles of the linear propagation of each probe beam. As shown in (Figs. 3$\hbox {c}_1$ and $\hbox {c}_2$), when we inject the probe beam in (7), we observe five outputs ($1^{\prime }$, $2^{\prime }$, $3^{\prime }$, $6^{\prime }$, $7^{\prime }$). In other words, this photo-induced configuration in the PR crystal permits transmitting the information from the source (7) to five destinations for all-optical routing applications. Similarly, as illustrated in (Fig. 3$\hbox {d}_1$ and $\hbox {d}_2$), the optical beam injected in (4) will be transmitted to four destinations ($6^{\prime }$, $7^{\prime }$, $8^{\prime }$, $11^{\prime }$). Then, as shown in (Fig. 3$\hbox {b}_1$ and $\hbox {b}_2$), we add another identical probe beam injected in the position between the main lobe (0) and the first side lobe (5) of the forward BB. Figure 3$\hbox {e}_1$ and $\hbox {e}_2$ depict the individual propagation of the adding probe beam. Compared to the (Fig. 2$\hbox {d}_2$ and $\hbox {d}_3$), due to the offset of the injecting position, the injected probe beam is transmitted not only to two main destinations ($10^{\prime }$, $11^{\prime }$) but also to the output ($6^{\prime }$). In this way, this waveguiding structure induced by two CP BBs allows us to send the information to nine destinations at the same time. In addition, it is possible to route the information in one specified channel as unicast routing by shifting the injected position of the probe beam. For example, Fig. 3$\hbox {c}_1$ and $\hbox {c}_2$ show the broadcast routing to five destinations when the probe beam is injected in position (7). Instead, as shown in (Fig. 3$\hbox {f}_1$ and $\hbox {f}_2$), if we move the injecting position down to (6), the probe beam is only guided to the output ($6^{\prime }$), and the guiding efficiency is high ($\approx 57 \%$). Therefore, we demonstrated that the waveguiding structure in (Fig. 2$\hbox {d}_1$) can simultaneously route the information from three sources to nine outputs that cannot be achieved by either Gaussian beams, Airy beams, or a single BB. Moreover, this photo-induced waveguiding configuration allows us to select the routing path and destination by shifting the injected position of the probe beam.

In addition, another advantage of using BBs results in their intrinsic parameter order *n* which can completely change their intensity profiles^31^, providing more possibilities for complex waveguiding structures. Interestingly, as our platform can independently tune the parameter of each incident beam, we can inject two BBs with different orders. Consequently, in the next part we investigate the influence of the beam profiles on the photo-induced waveguiding structures by changing the order parameters $n_1$ and $n_2$.

### More complex waveguiding structures through BBs’ orders interaction

**Figure 4:**
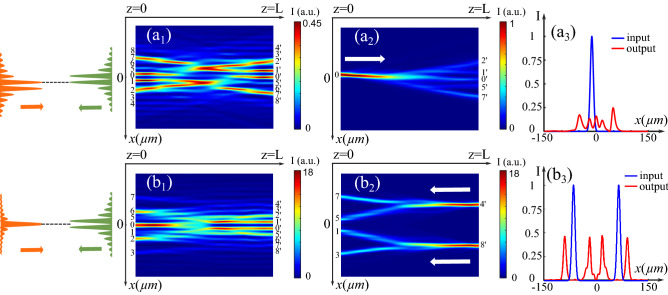
(${\mathbf{a}}_{{\mathbf{1}}}$) Normalized intensity distribution of the interaction of two CP one-order BBs ($x_0=10$ μm, $\omega _0=150$ μm, $F_0=B_0=\sqrt{1}$) with the transverse distance of $D=10$ μm under the nonlinear condition with $\Gamma = 6$. (${\mathbf{a}}_{{\mathbf{2}}}$) Intensity distribution and (${\mathbf{a}}_{{\mathbf{3}}}$) corresponding transverse profiles of linear probe beam propagation from (0) to ($0^{\prime }$, $1^{\prime }$, $2^{\prime }$, $5^{\prime }$, $7^{\prime }$). (${\mathbf{b}}_{{\mathbf{1}}}$) Normalized intensity distribution of the interaction of two CP aligned BBs with different orders (forward beam: $n=0,x_0=11$ μm, $\omega _0=150$ μm, $F_0=\sqrt{16}$; backward beam: $n=1,x_0=11$ μm, $\omega _0=150$ μm, $B_0=\sqrt{36}$) under the nonlinear condition with $\Gamma = 4$. (${\mathbf{b}}_{{\mathbf{2}}}$)(${\mathbf{b}}_{{\mathbf{3}}}$) Intensity distribution and corresponding transverse profiles of linear probe beam propagation from ($4^{\prime }$, $8^{\prime }$) to (1,3,5,7).

We firstly propagate two CP one-order ($n_1=n_2=1$) BBs with $x_0=10$ μm, $\omega _0=150$ μm, $F_0=B_0=\sqrt{1}$ under the nonlinear condition ($\Gamma =6$). Instead of the bright central spot of the zero-order BB, there is no light in the center of the high order BB. As illustrated by the principle scheme on the left of (Fig. 4$\hbox {a}_1$), we stagger them by a transverse distance of $10$ μm so that the top first lobe of the forward beam (0) aligns with the bottom first lobe of the backward beam ($0^{\prime }$). The intensity distribution of the nonlinear interaction of these two BBs is presented in (Fig. 4$\hbox {a}_1$). Compared to zero-order BBs, the intensity of one-order BBs decreases more slowly for high-order side lobes. Thus, the first few lobes with similar intensity excite almost the same amount of free charge carriers^6^. Such space-charge fields induce the refractive index changes with similar energy trapping capacity. Compared to the configuration induced by two zero-order BBs in (Fig. 1$\hbox {b}_1$), there is no channel with a relatively quite high refractive index change in the configuration in (Fig. 4$\hbox {a}_1$). As a result, as shown in (Fig. 4$\hbox {a}_2$ and $\hbox {a}_3$), when a probe beam is injected in 0, it propagates and splits in the middle of the crystal, yielding finally five outputs with roughly the same intensity at ($0^{\prime }$, $1^{\prime }$, $2^{\prime }$, $5^{\prime }$, $7^{\prime }$).

Subsequently, we inject two BBs with different orders: a zero-order BB ($n_1=0,x_0=11$ μm, $\omega _0=150$ μm, $F_0=\sqrt{16}$) as the forward beam and a one-order BB ($n_2=1,\;x_0=11$ μm, $\omega _0=150$ μm, $B_0=\sqrt{36}$) as the backward beam. Figure 4$\hbox {b}_1$ shows the principle scheme (on the left) and the intensity distribution of the interaction under the nonlinear condition ($\Gamma = 4$). The central dark spot of the backward beam is aligned with the main lobe of the forward beam, and the cross-coupling of the two beams generates parallel channels. To test the guiding efficiency of these channels, we inject two probe beams in ($4^{\prime }$, $8^{\prime }$) and propagate them to the (− *z*)-direction. Figure 4$\hbox {b}_2$ and $\hbox {b}_3$ show the intensity distribution and the transverse profiles. Both beams split into two outputs (7,5) and (1,3) with the identical intensity of $45\%$ of the maximum intensity. This coupler guides more than $90\%$ of the total energy, which is more efficient than those with two inputs and four outputs induced by a single second-order BB ($70\%$, $85\%$)^26^. Besides, as shown in (Fig. 4$\hbox {b}_3$), the distance between outputs (1) and (5) is approximately $3.2x_0$. The distance between outputs (7) and (5) is approximately $6.5x_0$ [same for (1, 3)]. It is worth noting that the outputs positions in this configuration are further apart than that discussed in^26^ ($\approx 2x_0$). Such large distances offer easier solutions for addressing applications.

So far, we have demonstrated that two CP BBs can induce waveguiding structures with multiple inputs and outputs in a biased PR crystal. Moreover, the BB truncation, the transverse distance, and the orders of the BBs are crucial parameters for tailoring the photo-induced waveguiding structures via refractive index modulations. It is worth noting that all previously discussed configurations concern the stationary state after a short transient regime. As analyzed in^12^ and^32^, the steady-state is not always reached in the cases of two interacting CP beams but may present a peculiar spatio-temporal behavior related to the nonlinearity $\Gamma$ in the system. To complete our study, we decide to test the impact of the nonlinearity over the spatiotemporal dynamics of two CP BBs.

### Nonlinearity inducing peculiar spatiotemporal dynamics

**Figure 5:**
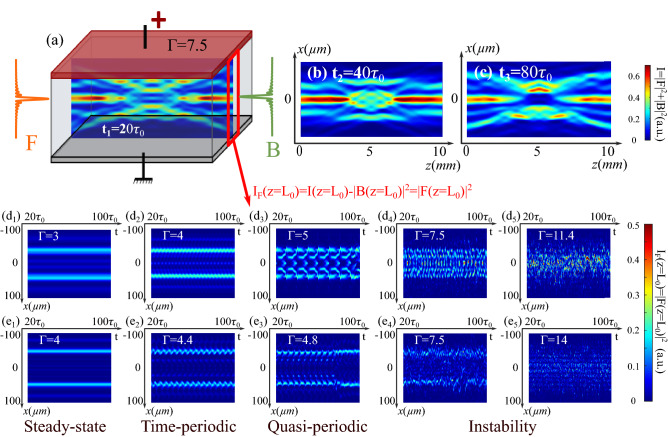
(**a**)–(**c**) Waveguiding structures induced by two zero-order BBs ($x_0=10$ μm, $\omega _0=150$ μm, $F_0=B_0=\sqrt{25}$) under the nonlinear condition of $\Gamma =7.5$ at different instants: (**a**) $t_1=20\tau _0$ (**b**) $t_2=40\tau _0$ (**c**) $t_3=80\tau _0$ (${\mathbf{d}}_{{\mathbf{1}}}$)–(${\mathbf{d}}_{{\mathbf{5}}}$) Temporal evolution of the transverse output positions of the forward beam at $z=L$ in the case of two zero-order BBs ($x_0=10$ μm, $\omega _0=150$ μm, $F_0=B_0=\sqrt{25}$): (${\mathbf{d}}_{{\mathbf{1}}}$) steady-state ($\Gamma$ = 3), (${\mathbf{d}}_{{\mathbf{2}}}$) periodical and symmetrical oscillations ($\Gamma$ = 4), (${\mathbf{d}}_{{\mathbf{3}}}$) quasi-periodical state ($\Gamma$ = 5), (${\mathbf{d}}_{{\mathbf{4}}}$) instability ($\Gamma$ = 7.5), and (${\mathbf{d}}_{{\mathbf{5}}}$) instability ($\Gamma$ = 11.4). (${\mathbf{e}}_{{\mathbf{1}}}$)–(${\mathbf{e}}_{{\mathbf{5}}}$) Temporal evolution of the transverse output positions of the forward beam at $z=L$ in the case of two one-order BBs: (${\mathbf{e}}_{{\mathbf{1}}}$) steady-state ($\Gamma$ = 4), (${\mathbf{e}}_{{\mathbf{2}}}$) periodical and symmetrical oscillations ($\Gamma$ = 4.4), (${\mathbf{e}}_{{\mathbf{3}}}$) quasi-periodical state ($\Gamma$ = 4.8), (${\mathbf{e}}_{{\mathbf{4}}}$) instability ($\Gamma$ = 7.5), and (${\mathbf{e}}_{{\mathbf{5}}}$) instability ($\Gamma$ = 14).

In this section, we keep the same beam parameters as those in (Fig. 2) and set two CP BBs facing each other and perfectly aligned ($D=0$). The stationary waveguiding structure induced under the nonlinear condition of $\Gamma = 3$ has been shown and discussed in (Fig. 2$\hbox {a}_1$). This time, as shown in (Fig. 5a), we set a high nonlinearity strength: $\Gamma =7.5$. The intensity distributions depicted in (Fig. 5a–c) respectively present the waveguiding configurations formed at the instants $t_1=20 \tau _0$, $t_1=40 \tau _0$, $t_1=80 \tau _0$. The waveguiding structure changes over time without regularity such as it is hard to predict the waveguiding configuration at a given time from a previous instant. Considering this result, we will analyze in what follows the time-dependent behavior of the waveguiding structures under different nonlinear conditions by changing the $\Gamma$ parameter. For each $\Gamma$ value, we calculate the interaction of two CP BBs up to $100\tau _0$ in a step of $1/15{\tau _0}$. For a more visual comparison, in each time step, as indicated in (Fig. 5a), we extract the output intensity of the forward beam *F* using $I_F(z=L_0)=|F(z=L_0)|^2$ and plot the output intensity distribution over time in (Fig. 5$\hbox {d}_1$–$\hbox {d}_5$). To avoid the short transient period, we only cover the period between $20 \tau _0$ and $100 \tau _0$ in these figures. Thus, Fig. 5$\hbox {d}_1$–$\hbox {d}_5$ display the temporal fluctuations of the transverse output positions of the forward beam *F* in the zero-order cases. Figure 5$\hbox {d}_1$ is the result calculated under $\Gamma =3$, corresponding to the stationary waveguiding structure in (Fig. 2$\hbox {d}_1$). The two most intense outputs in (Fig. 5$\hbox {d}_1$) correspond to positions ($2^{\prime }$, $6^{\prime }$) in (Fig. 2$\hbox {d}_1$), where the two branches of the Y-coupler induced by the forward beam overlap with the side lobes of the backward beam. When increasing $\Gamma$ to 4, as shown in (Fig. 5$\hbox {d}_2$), the time trace along *x* exhibits a time-periodical evolution with a period of $3.4 \tau _0$. Then, as the nonlinearity increases, for example, for $\Gamma =5$ in (Fig. 5$\hbox {d}_3$), a transverse symmetry-breaking arises after the periodic evolution at around $t=90 \tau _0$, resulting in irregular spatiotemporal dynamics, the so-called quasi-periodical state. When $\Gamma >6.5$, the two beams move and interact erratically so that the configuration reaches chaotic-like state. As depicted in (Fig. 5$\hbox {a}_4$), when $\Gamma =7.5$, corresponding to the case shown in (Fig. 5a–c), despite irregular sequences versus times, the positions are concentrated in the zone corresponding to the lobes of the backward beam. When the nonlinearity still increases [$\Gamma =11.4$ in Fig. 5$\hbox {a}_5$], the distribution of the output positions no longer has any regularity.

As mentioned in the last section, the order of each incident BB influences the CP scheme. Therefore, we consider another case that two incoherent one-order BBs ($n_1=n_2=1$) propagate without misalignment. We set the same beam parameters as before ($x_0=10$ μm, $\omega _0=150~\mu m$ μm, $F_0=B_0=\sqrt{25}$) and plot the output intensity distributions over time (Fig. 5$\hbox {e}_1$–$\hbox {e}_5$) in the same way. In this case, we also identify four stages for the spatiotemporal evolution of the CP beams interaction: the stationary state (as shown in Fig. 5$\hbox {e}_1$) for $\Gamma =4$), the periodical oscillation dynamics (as shown in Fig. 5$\hbox {e}_2$ for $\Gamma =4.4$), the quasi-periodical state with symmetry-breaking instabilities (as shown in Fig. 5$\hbox {e}_3$ for $\Gamma =4.8$), the unstable state (as shown in Figs. 5$\hbox {e}_4$ and $\hbox {e}_5$ for respectively $\Gamma =7.5$ and $\Gamma =14$).

Furthermore, it is worth noting that, compared to the case of the zero-order beams in (Fig. 5$\hbox {d}_5$), above $\Gamma =7.5$, as shown in (Fig. 5$\hbox {e}_5$), the output positions are arranged and concentrated in the positions corresponding to the backward beam lobes ($\Gamma =14$) in the case of two one-order BBs.

**Figure 6:**
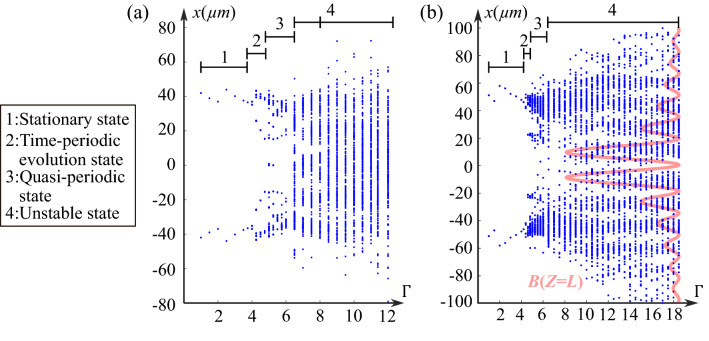
(**a**) Bifurcation diagram of the transverse output positions of the forward beam at $z=L_0$ in the case of two CP zero-order BBs. (**b**) Bifurcation diagram of the transverse output positions of the forward beam at $z=L_0$ in the case of two CP one-order BBs.

Thus, to specify the dynamic behavior of the CP interactions versus nonlinearity in the previous two cases, as discussed, we plot the x-extrema of the forward beam’s outputs for different $\Gamma$ values (Fig. 6a and b). Same as previous calculations, for each $\Gamma$, we simulate the propagation until $t=100\tau _0$ and only consider the output positions of *F* within the $20 \tau _0$ to $100 \tau _0$ for plotting the diagrams. The diagram displays the route to instabilities starting from a system with a weak nonlinearity ($\Gamma < 4$) to a highly nonlinear system ($\Gamma > 10$). For the zero-order case, when $\Gamma <4$, only one point is plotted on each side of $x=0$ on (Fig. 6a), which corresponds to the *x*-values of the stationary two output positions (Fig. 5$\hbox {d}_1$). When $\Gamma \in [4,4.8]$, the distribution of the extreme points is strictly symmetric about $x=0$, corresponding to the time-periodic dynamics. For $\Gamma \in [4.9,6]$, the distribution is no longer symmetrical because of the symmetry-breaking identified in (Fig. 5$\hbox {d}_2$–$\hbox {d}_3$). Then for $\Gamma \in [6.5,8]$, the points are located in the positions of the backward beam lobes, which corresponds to the unstable state as (Fig. 5$\hbox {d}_4$). Finally, when $\Gamma \geqslant 8.5$, for each $\Gamma$-value, the points are distributed on a line showing a chaotic-like behavior.

Similarly, we can identify spatiotemporal dynamic stages corresponding to that shown in (Fig. 5$\hbox {e}_1$–$\hbox {e}_5$) in the bifurcation diagram (Fig. 6b): $\Gamma \leqslant 4.2$ for stationary state, [4.4, 4.6] for time-periodic dynamics, [4.8, 6] for quasi-periodic oscillations state, and $\Gamma \geqslant 6.5$ for two types of unstable states. More interestingly, for two one-order BBs, under very high nonlinearity, we observe that all output positions are distributed discretely in the zone of the backward beam’s lobes which corresponds to the phenomenon in (Fig. 5$\hbox {e}_5$). As shown in (Fig. 6b), it is more probable that the forward beam outputs locate in the first few order lobes of the CP BB than in its high-order lobes. As discussed in^32^, this phenomenon indicates that the attraction strength decreases for the higher lobe orders because of the gradually decreasing space-charge field related to the energy distribution.

Therefore, by analyzing the spatiotemporal behavior of the CP incoherent zero-order and first-order BBs, we identified the thresholds for the non stationary states in both cases: $\Gamma =4$ for the zero-order BBs, $\Gamma =4.4$ for the one-order BBs. Also, in both two cases, by increasing the nonlinearity strength $\Gamma$, we demonstrated four different regimes: stationary state, time-periodic evolution state, quasi-periodic state, and unstable state. We conclude that the nonlinearity can tailor the temporal behavior of the waveguides induced by two CP BBs. In addition, we observe the spatially-localized distribution of the instabilities for higher order BBs, which suggests that the interaction pattern is controllable by the CP BB (backward BB in the previous case) even though in the chaotic-like regime. The above results provide new techniques for active optical components, such as dynamically varying waveguides, in all-optical communication applications.

## Conclusion

In conclusion, we numerically investigated the photo-inscription of waveguiding structures by two incoherent CP BBs in a biased PR crystal. Firstly, compared with the 1D waveguides induced by one single BB^26^ and two CP Gaussian beams^7^, the cross-coupling between two CP BBs enables more accessible channels, high guiding efficiencies, and a large transverse dimension of the photo-induced waveguiding structures (up to $8x_0$ for the transverse input-to-output shift). Compared to the CP interactions of the Airy beams^12,14^, the optical splitter created by two BBs owns more addressable outputs (up to five outputs). Secondly, by changing the parameters, such as the Gaussian truncation ($\omega _0$), the misalignment between the two CP BBs (*D*), and the order of each incident BB ($n_1$, $n_2$), we can completely control the number of the outputs, the output intensity levels (via the guiding efficiency of each channel), and the distance between each output. Finally, we analyzed the spatiotemporal dynamics of the CP interactions of two incoherent zero-order BBs or two incoherent one-order BBs. We identify the stability thresholds for both cases: $\Gamma = 4$ for the zero-order BBs and $\Gamma = 4.4$ for the one-order BBs. Above these thresholds, the steady-state bifurcates to time-periodic, quasi-periodic and unstable states. When two incoherent CP high-order BBs interact under very high nonlinear conditions, the outputs of the forward beam irregularly move around the positions corresponding to the backward beam’s lobes. This lobe-chosen behavior suggests that the backward beam can control the output positions of the forward beam in the chaotic-like state. All the above discussions enlarge further possibilities for all-optical interconnects and motivate the technologies of fully reconfigurable optical routing as well as active optical components for all-optical telecommunication. It is worth noting that more phenomena may be caused, for instance, by the anisotropy of the crystal and/or the 2D profiles of Bessel beams that could be observed only in a 3D configuration. Our simulations using a (1+1)D numerical model achieve, therefore, the first step to induce complex waveguiding structures in a 3D PR crystal and motivate new (2+1)D simulations for future works.

## Data Availability

Te datasets used and/or analysed during the current study are available from the corresponding author on reasonable request.
